# Signs and Symptoms of Acoustic Neuroma at Initial Presentation: An Exploratory Analysis

**DOI:** 10.7759/cureus.1846

**Published:** 2017-11-15

**Authors:** Robert W Foley, Shahram Shirazi, Robert M Maweni, Kay Walsh, Rory McConn Walsh, Mohsen Javadpour, Daniel Rawluk

**Affiliations:** 1 Department of Neurosurgery, Addenbrooke's Hospital, Cambridge University Hospitals, United Kingdom; 2 School of Medicine, University College Dublin; 3 Department of Skull Base Surgery, Beaumont Hospital, Dublin, Ireland; 4 Department of Otolaryngology, Beaumont Hospital, Dublin, Ireland; 5 Department of Neurosurgery, Beaumont Hospital, Dublin, Ireland

**Keywords:** acoustic neuroma, diagnosis, epidemiology, signs and symptoms

## Abstract

Introduction

The objective of this study was to describe the most common clinical features associated with an acoustic neuroma diagnosis and to identify those features associated with larger tumour size at initial diagnosis.

Methods

The clinical information of 945 consecutive patients diagnosed with acoustic neuroma at a single centre between 1992 and 2015 was analysed. Clinical features were examined and the relationship between these features and tumour size (>2.5 cm) was analysed using descriptive statistics and logistic regression analysis. Statistical analysis was performed in R version 3.1.1.

Results

The most common presenting symptom was a unilateral hearing loss in 752 patients (80%), with a progressive pattern in 90% of these cases. The second most common presenting symptom was unilateral tinnitus, accounting for 6.3%, while ataxia, vertigo and headache accounted for 3.8%, 3.4% and 2%, respectively. The diagnosis of acoustic neuroma was an incidental finding in 20 patients (2.1%). Temporal analysis demonstrated a downward trend in the number of patients presenting with hearing loss and an increased proportion of patients presenting with other symptoms. On multivariate analysis, larger tumour size was associated with abnormal tandem gait (odds ratio 8.9, p=0.02), subjective facial weakness (odds ratio 5.3, p< 0.001), abnormal facial sensation on examination (odds ratio 3.0, p=0.03) and headache (odds ratio 2.6, p< 0.001).

Conclusion

The majority of patients with acoustic neuroma present with the classic, progressive, unilateral hearing loss. However, the pattern of presentation in acoustic neuroma patients is changing. Features in the history indicative of a larger tumour are headaches and subjective facial weakness, whilst concerning features on examination are abnormal tandem gait and altered facial sensation.

## Introduction

Acoustic neuroma, or vestibular schwannoma, is a benign tumour of the Schwann cell that most commonly arises from the vestibular nerve [1]. Acoustic neuroma is one of the most common intracranial tumours and has been reported to make up approximately 6%-10% of tumours in most series [2]. Acoustic neuroma has an incidence of approximately 12 per million in the United States of America and approximately 20 per million in a contemporary European cohort [3-4]. However, the incidence of acoustic neuroma is increasing [5-6]. An analysis of 26 years worth of prospectively collected data, from 1976 to 2002 in Denmark, has shown the incidence move from 7.8 per million to 19.3 million [5]. In tandem with these changes, there are increasing numbers of smaller tumours being diagnosed. The Danish data has shown a size decrease from an average of 30 mm to an average of 10 mm in the period from 1976 to 2008. These trends in the epidemiology of acoustic neuroma may mean that the presentation of patients suffering from this disease may also be changing. Thus, it would be of benefit for healthcare providers, be they in primary care, a hospital setting or a surgical sub-specialty, to be aware of any emerging patterns in the presentation of acoustic neuroma patients. Descriptions of the signs and symptoms of acoustic neuroma within the literature are largely from older studies, which may not be applicable to contemporary practice [7-8]. Furthermore, adequate statistical analysis of these variables with multivariable regression has yet to be performed.

Therefore, the objectives of this study were to describe the most common clinical features associated with the initial presentation of patients with acoustic neuroma and to examine the temporal trends in these features. Within the acoustic neuroma management paradigm, larger tumours are an important clinical entity, as they require earlier definitive treatment. The study of clinical and historical features within each patient’s presentation and the identification of the features associated with larger tumours will allow for an accurate diagnosis to be made quickly and, therefore, the appropriate investigations can be targeted towards those patients with the greatest clinical need. Hence, a secondary objective of this study was to identify those clinical features associated with larger tumour size at initial diagnosis.

## Materials and methods

All patients within the cohort were diagnosed with acoustic neuroma and their data collected prospectively and added to a database. The present study analyses the data of all patients within the database from its inception in 1992 until 2015. Each patient was referred to the national neurosurgical centre at Beaumont Hospital, Ireland, and underwent a thorough history and examination, which was recorded in the database. Clinical features in the history and examination are reported by the number of patients and the percentage, with all percentages rounded to the nearest whole number. Tinnitus severity was recorded as mild, moderate or severe, as per Baguley et al. [9]. Facial nerve function was determined using the House-Brackmann grading scale [10]. Tumour size was measured based on each patient’s initial magnetic resonance imaging (MRI) scan and is the extrameatal maximum diameter of the tumour. Size was recorded in one of five categories, namely, <1.5 cm, 1.5-2.4 cm, 2.5-3.4 cm, 3.5-4.4 cm and >4.5 cm.

In order to analyse those patients with larger tumours, tumour size was dichotomised as those less than 2.5 cm and those 2.5 cm or greater. The relationship between patient clinical characteristics and tumour size was analysed by univariable and multivariable logistic regression. Odds ratios (OR) and 95% confidence intervals were calculated and a p value of <0.05 is considered statistically significant. Time series analysis took place via the statistical package “TTR” and data was smoothed using simple moving averages. A statistical analysis was carried out using R v0.98 and the relevant statistical packages.

## Results

The study cohort consisted of 945 consecutive patients diagnosed with acoustic neuroma. In this cohort, 752 patients (80%) presented with unilateral hearing loss. The majority presented with a progressive hearing loss, which accounted for 90% of these patients. A sudden unilateral hearing loss was the second-most common overall presenting complaint, with 72 patients presenting in this way. Tinnitus was the main complaint in 60 patients (6%). Acoustic neuroma was an incidental finding in 20 patients within this cohort. The other presenting complaints and the proportion of patients are demonstrated in Table 1.

**Table 1:** Main presenting symptom in the total cohort (n=945) The number of patients presenting with each symptom - as the main presenting complaint - is illustrated, as is the percentage this represents within the total cohort.

	n	%
Hearing Loss	752	80
- Progressive Hearing Loss	680	72
- Sudden Hearing Loss	72	8
Tinnitus	60	6
Ataxia	36	4
Vertigo	32	3
Asymptomatic / Incidental	20	2
Headache	19	2
Facial Numbness	17	2
Otalgia	2	<1
Facial Pain	1	<1
Seizure	1	<1
Syncope	1	<1

In terms of overall symptoms that patients were suffering from, not only the main presenting symptom, hearing loss was present in 868 patients (92%), with 91% complaining of a progressive course. A total of 479 patients (51%) complained of tinnitus, 388 patients (81%) intermittently and 91 patients (19%) with constant symptoms. In terms of tinnitus severity, 330 patients (68%) suffered from mild tinnitus, 144 (30%) from moderate and 11 (2%) from severe tinnitus. Headache was a historical feature in 114 patients (12%), with 61% of these generalised and 39% focal in location. A separate pain was described in 40 patients, with a mastoid ache in 32 and otalgia in eight patients. A total of 234 patients (25%) complained of unsteadiness when questioned. Other symptoms included facial weakness in 75 patients, facial numbness in 26 patients, blurred vision in 20 patients and dry eye in five patients. Altered sensations of the tongue and of taste were present in 15 and 13 patients, respectively. On examination, the vast majority of patients, 916 (97%), were House-Brackmann grade I, with 20 grade II patients, two grade III, two grade IV, one grade V and four grade VI. An abnormal trigeminal sensation on examination was present in 29 patients. An examination of the fundi revealed mild disc blurring in eight patients. Gait testing revealed abnormalities of tandem gait in 54 patients (6%).

**Figure 1:**
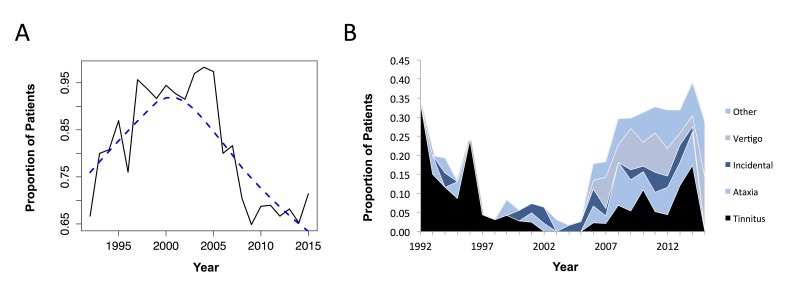
Trends in initial presentation with regards to unilateral hearing loss (A) and primary presenting symptom (B) (A) The proportion of patients presenting with hearing loss as the main presenting symptom is shown on the x-axis (e.g., 0.75 corresponding to 75% of patients). The blue dashed line shows the smoothed pattern over time following a time series analysis. (B) The trend in presenting symptoms other than hearing loss. This demonstrates that in the later years of the study (right side of the graph), there has been an increase in patients presenting with ataxia, vertigo and incidental tumours.

Over the 23 years of the study period, there have been changes in the pattern of patient presentation. The proportion of patients complaining of hearing loss as the main presenting feature, overall, is decreasing (Fig. 1A). There is a clear downward trend for presentation with hearing loss in acoustic neuroma patients in the last decade. Whilst hearing loss accounted for the vast majority of presentations in the late 1990s and early 2000s, since then there has been increasing variability in the presenting symptom, with ataxia, vertigo and incidental findings accounting for more patient’s presentations (Fig. 1B). The number of patients in Ireland with acoustic neuroma is increasing steadily each year, with 20 patients on average at the start of the study period, moving to greater than 60 patients each year in the later years. The average period from symptom onset to clinical presentation was 36 weeks in this study cohort. A time series analysis demonstrated no trend in time to presentation over the course of the study period. However, patients with sudden hearing loss presented in an average of 12.1 weeks, which was significantly faster (p <0.01) than those with a progressive hearing loss, who presented on average within 45.6 weeks.

**Figure 2:**
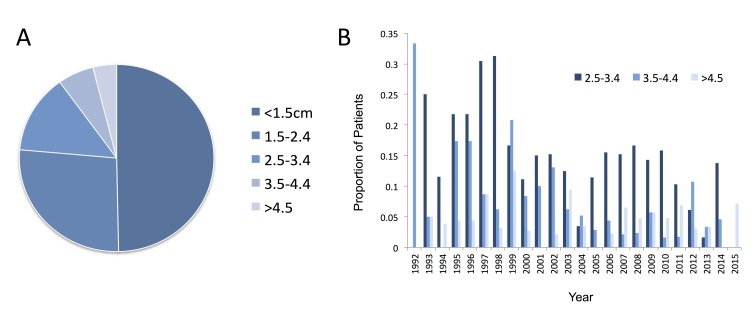
Tumour size in the total cohort (A) and the trend in the proportion of tumours within the larger size categories (B) The breakdown of larger tumour sizes (i.e., >2.5 cm) is shown in (B), with a decreasing trend in these larger tumours evident over the study period.

Tumour size within the study cohort was split into four categories, as illustrated in Fig. 2A. Tumours less than 1.5 cm in size accounted for 48% of all tumours, with 25% between 1.5-2.4 cm, 13% 2.5-3.4 cm, 5% 3.5-4.4 cm and 4% greater than 4.5 cm. The trend in tumour size at diagnosis is clear in this cohort. The number of smaller tumours (<2.5 cm) is increasing, while those >2.5 cm in size are decreasing year on year (Fig. 2B). Subsequently, fewer patients were treated with a primary surgical treatment strategy, with >80% on average treated surgically at the beginning of the study period, compared to <20% in the last seven years of the study period.

A univariable and multivariable regression analysis was undertaken to examine the clinical features most associated with a larger tumour size at diagnosis. A large number of factors was associated with larger tumour size on univariate analysis. This included taste alteration (OR 5.34, 95% CI: 1.76-17.83; p<0.01), visual alterations (OR 9.60, 95% CI: p<0.01), a positive Romberg’s test (OR 6.05, 95% CI: 2.07-19.9; p<0.01) and dysdiadochokinesis (OR 6.31, 95% CI: 2.55-16.99; p<0.01). However, there were only four features that were significant predictors of tumour size on both univariable and multivariable analyses (Table 2). These are both historical features and examination features. On a multivariate analysis, larger tumour size was associated with abnormal tandem gait (OR 8.9), subjective facial weakness (OR 5.3), abnormal facial sensation on examination (OR 3.0) and the presence of headache (OR 2.6). Factors that did not discriminate between smaller and larger tumour size were the presence of hearing loss (OR 0.98, p=0.95), sudden versus progressive pattern of hearing loss (OR 0.93, p=0.82), time until presentation (OR 0.99, p=0.14) and severity of tinnitus (OR 2.12, p=0.68).

**Table 2:** Odds ratios and 95% confidence intervals for the association of various patient characteristics and a large tumour size (≥2.5 cm) † Multivariate analysis also controlling for age, hearing loss presence, pattern of hearing loss, tinnitus presence, tinnitus severity, weeks from symptom onset to presentation and all other historical features or neurological examination findings*.*

	Univariate Analysis		Multivariate Analysis^†^	
	Odds Ratio (95% CI)	p value	Odds Ratio (95% CI)	p value
Historical Features				
Headache	6.26 (4.13-9.56)	<0.001	3.11 (1.79-5.40)	<0.001
Facial Weakness	8.85 (5.31-15.16)	0.18	5.25 (2.66-10.57)	<0.001
Examination Features				
Tandem Gait (eyes closed)	9.18 (4.99-17.68)	0.97	8.94 (1.35-71.92)	0.022
Altered Trigeminal Sensation	10.28 (4.71-24.85)	<0.001	3.04 (1.08-8.78)	<0.001

## Discussion

Acoustic neuroma is an important clinical entity, and the number of patients presenting with this condition is increasing. The diagnosis of acoustic neuroma is nearing 20 new cases per million population, per year [5-6]. In the 23 years of the present study, the annual numbers of acoustic neuroma diagnoses have tripled. Kleijwegt et al. have found a similar increase in diagnosis over a ten-year period [11], and Stangerup et al. described a near 400% increase in patients being diagnosed over a 25-year period [5]. In tandem with the increasing number of patients, this study has demonstrated a downward trend in the numbers of patients presenting with larger tumours. This trend in diagnosis has meant that there are larger numbers of patients presenting with smaller tumours and there has been a steady decrease in surgically treated patients at our centre, in keeping with previously published findings [4,12-14]. As has been seen worldwide, the trend in acoustic neuroma management is to utilise a more conservative approach, with increasing numbers of patients under observation or treated with radiosurgery, and surgery now focusing on preservation of the facial nerve [12].

A large multi-national survey-based study identified unilateral hearing loss in 86% of patients, with unsteadiness in 61%, tinnitus in 57% and headache in 36% [7]. However, this study from Wiegand et al. does not draw comparisons between these symptoms and patient tumour size. Nor does this particular study differentiate between the main presenting symptoms or discuss symptom severity. The contemporary series of patients outlined in this article showed similar levels of hearing loss and tinnitus, but smaller numbers of positive cranial nerve abnormalities and cerebellar signs. The explanation for this is likely because hearing loss and tinnitus are not size-dependent features of acoustic neuroma, whilst symptoms and signs associated with compression of the nearby cranial nerves, brainstem and cerebellum are size dependent. Matthies et al. provide a series of 1,000 patients and explain that 95% of patients undergoing surgery have hearing loss prior to their operation and 63% were suffering from tinnitus [15]. The authors go on to report vestibular disturbances in 61%, headaches in 12% and taste disturbances in 2%. However, there is no analysis of the relationship of these symptoms to tumour size and, furthermore, these historical and clinical features are not at presentation but rather at the time of surgery and, so, comparison with the results of the present article are difficult.

In this study, the data trends indicate a decreasing number of patients presenting with the classical complaint of unilateral hearing loss. Tinnitus, as the second-most common presenting symptom, has been relatively stable over the years of the study, accounting for 5%-10% of presenting complaints overall. Other presentations, such as ataxia, vertigo and incidental findings, are becoming increasingly common. Other presentations that are emerging include altered facial sensation, facial pain, headache, otalgia and syncope. One possible explanation for this is that the availability of MRI is allowing these patients to be investigated more thoroughly, even with atypical symptoms [6]. Another explanation could be that in a modern, medicalised society, patients are more likely to present to their physician with a wider array of symptoms and at an earlier stage than in the past. Although the pattern of presentation appears to be changing, the vast majority of patients do still have one or more of the classic symptoms of acoustic neuroma. On direct questioning of all possible symptoms, in a cohort of 473 patients, Moffat et al. found hearing loss, tinnitus or imbalance in 85%, 73% and 66% of patients, respectively [8]. This demonstrated that the majority of patients suffered from one or more of the classic acoustic neuroma symptoms prior to their diagnosis. In fact, only two out of their 473 patients experienced none of these symptoms. In the present study, 21 patients experienced none of these classical symptoms. The most common symptom experienced in this atypical group was facial numbness (24%). A case series (n=9) of atypical acoustic neuroma presentations also found that facial numbness was the most common presenting symptom in 22% of patients [16]. Clinicians should, therefore, be aware of this as a potential, albeit unusual, presenting symptom of acoustic neuroma. In other words, in the absence of the classic disturbance to the vestibulocochlear nerve, the next most-common disturbance is of trigeminal nerve function. Interestingly, no patient in our cohort presented with altered facial nerve function without also suffering from vestibulocochlear nerve dysfunction.

Tinnitus is a significant symptom in acoustic neuroma patients. The relationship between tinnitus and tumour size was investigated by Baguley et al [17], who demonstrated that patients with larger tumour sizes were less likely to experience tinnitus, and this finding was statistically significant [17]. This echoes our own results on univariate analysis. However, the authors did not go on to investigate this relationship on multivariate analysis, taking into account all other symptoms. On multivariate analysis in our cohort of patients, tinnitus presence or absence was no longer a statistically significant predictor of tumour size. Although not analysed in the present study, the duration of tinnitus prior to diagnosis has also been demonstrated in a large case series to be inversely related to tumour size, with longer duration of tinnitus associated with smaller tumours [15].

The incidence of sudden hearing loss in acoustic neuroma is variable and is reported in the literature with incidences from 5% to 22% [18]. The incidence of sudden hearing loss was 12% in a case series from Moffat et al. [18], with 85% complaining of this as the main presenting symptom. Sudden hearing loss was found in 76 patients (8%) within the present article’s cohort, and 72 of these patients complained of this as their principal presenting symptom. This finding was relevant, as it was associated with a shorter time to presentation compared to those patients with progressive hearing loss. In the paper from Moffat et al., the average time to presentation was 32 months in the sudden hearing loss group compared to 40 months in those with progressive hearing loss. These findings are supported by this study, with patients suffering from sudden hearing loss presenting significantly quicker than those with a progressive pattern. The length of time to presentation is an important factor in tumour size, as longer length of symptom duration is associated with larger tumours [7]. As with Moffat et al., there was no association found between hearing loss pattern and tumour size in the present study.

Moffat et al. have reported ten percent of patients with acoustic neuroma as having an atypical main presenting symptom, i.e., not presenting with hearing loss, imbalance or tinnitus [8], much the same figure as in the present study. Patients with these atypical presentations were also reported to be more likely to have larger tumours, perhaps because of a delay in investigation [8]. This finding was not replicated in the present study, which may reflect the increasing availability of MRI, and indeed a lower threshold for MRI use. The number of patients with tumour sizes of 2.5 cm or greater was much larger in the Moffat et al. cohort, at 45%, compared to 22% in our Irish cohort.

The number of incidental findings of acoustic neuroma appears to be low. Lin et al. [19] carried out an analysis of 505 patients and suggested that only eight of these patients underwent an incidental diagnosis. This equates to a prevalence of incidental acoustic neuromas of 1.6%, which is in keeping with the prevalence in the present article. Our study revealed an incidental diagnosis in 20 patients of the 945 patients, for a prevalence of 2.1%. The literature on this topic has identified a prevalence of incidental acoustic neuroma, ranging from 0%-2.4% [20]. Importantly, the number of patients being diagnosed with acoustic neuroma incidentally appears to be increasing. In the present study, half of the incidental cases had occurred in the last six years of the study period. This increasing number of incidental tumours represent an important clinical entity because although these tumours are generally smaller and require less intervention than symptomatic tumours [21], their growth pattern and natural history are still not elucidated. Indeed, some of the “incidental” findings of acoustic neuroma published elsewhere were found to have long-standing symptoms when questioned further [19]. In the present study, eight of the 20 incidental findings were found to have symptoms of hearing loss, tinnitus or imbalance on further questioning. Thus, the approximation for the incidence of truly atypical presentations in this cohort is 1.2% and, therefore, the number of patients with no experience of the typical symptoms of acoustic neuroma is very small. This emphasises the important role of clinical history, particularly within the primary care setting.

We recognise some limitations to this study. Only the original clinical presentation of these patients has been analysed and we have not examined the trends in these symptoms over time. Also, the tumour size presented in this study is the size at original presentation and, therefore, again, changes in size over time have not been measured. Furthermore, with regard to the self-reported length of time that symptoms have been present before presentation, this variable is subject to recall bias. One interesting variable, which may be in some way responsible for the increasing number of acoustic neuroma diagnoses, is the changing demographics of the population of Ireland. Within the timespan of the present study, the population of Ireland has grown considerably and the population is less ethnically homogenous than in the past. Unfortunately, racial ethnicity was not a recorded variable and could not be analysed.

## Conclusions

The majority of patients with acoustic neuroma present with the classic, progressive, unilateral hearing loss. Atypical presentations and incidental findings are becoming more common, highlighting the need for a thorough history and physical examination. Features in the history indicative of a larger tumour size at diagnosis are headache and subjective facial weakness, whilst concerning features on examination are abnormal tandem gait and altered facial sensation.
